# Supplementary material for: Heritable DNA methylation marks associated with susceptibility to breast cancer
Source: Nat Commun. 2018 Feb 28;9:867. doi: 10.1038/s41467-018-03058-6 (PMC5830448; doi:10.1038/s41467-018-03058-6)
Supplement: Supplementary file 1 — Supplementary Information [file 41467_2018_3058_MOESM1_ESM.pdf]

Supplementary Information

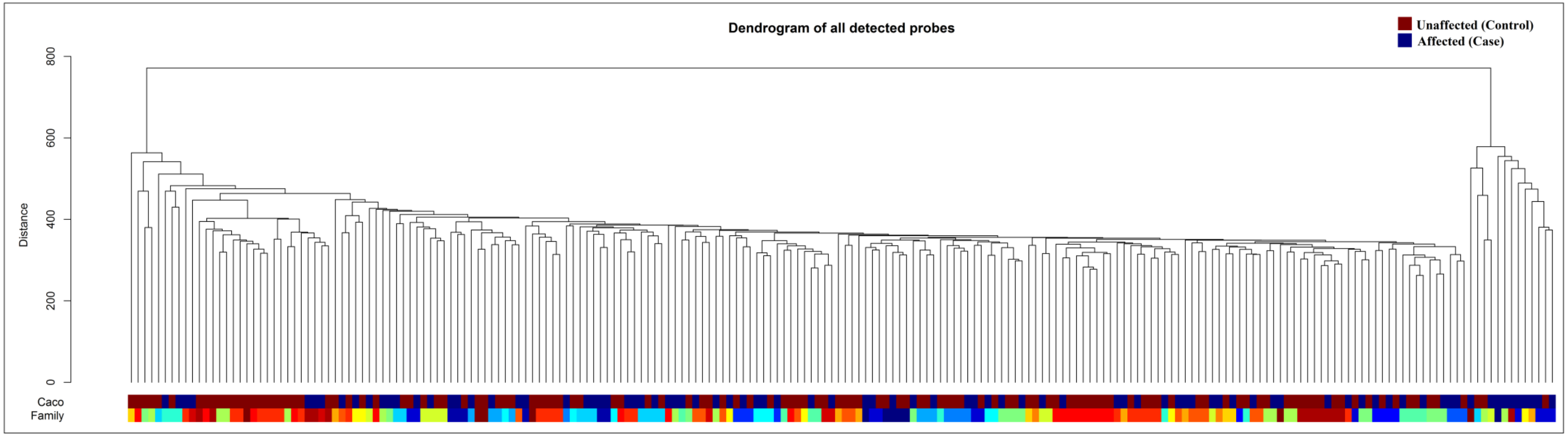

Supplementary Figure 1

Hierarchical clustering analysis of all samples and all 481,563 detected probes. Cluster analysis shows that there are no batch effects and that all technical replicates tend to cluster close to each other. Some DNA Methylation similarities were shared within some families. Each individual has been color-coded by family and affected status (Caco).

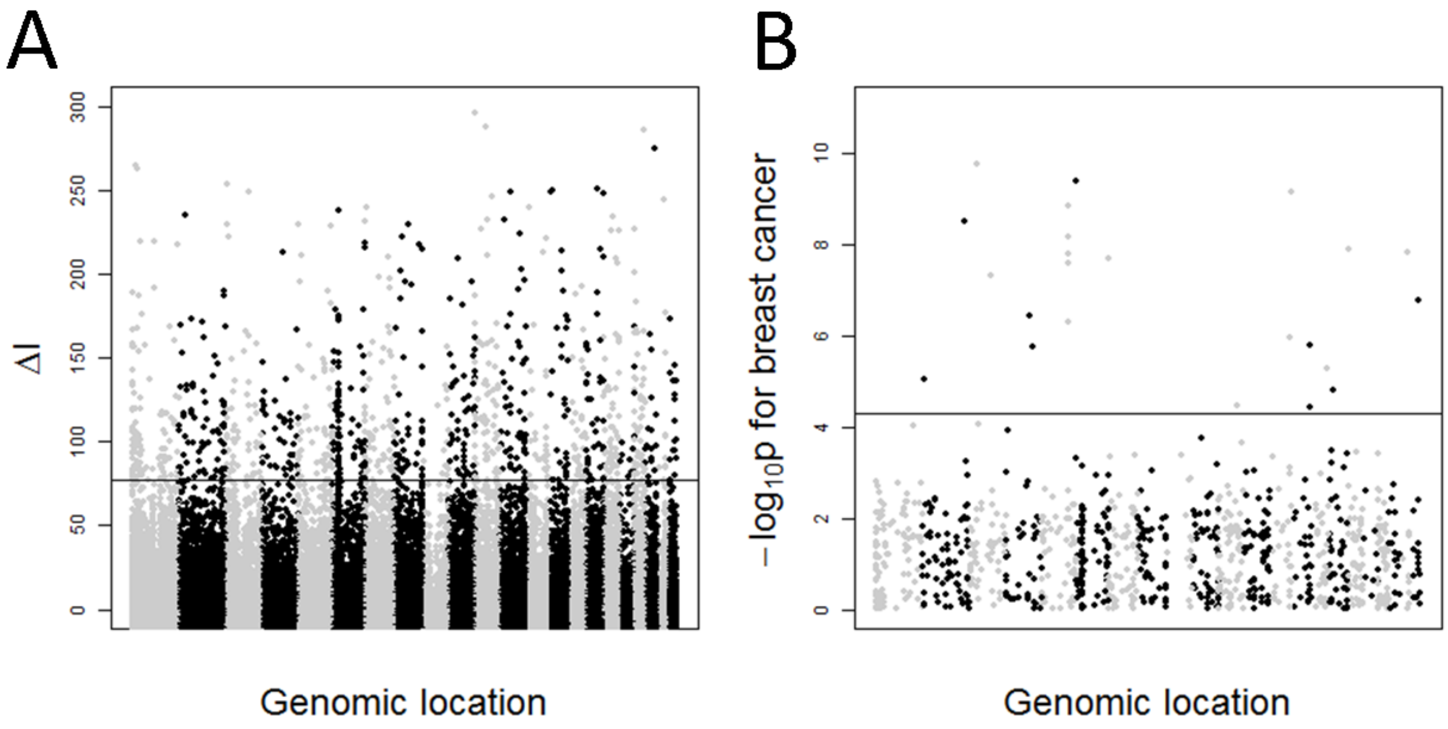

Supplementary Figure 2
Manhattan plots showing the statistic Δ*l* by chromosomal location for all sites (A) and the association with breast cancer by chromosomal location for the 1,000 most Mendelian sites (B).

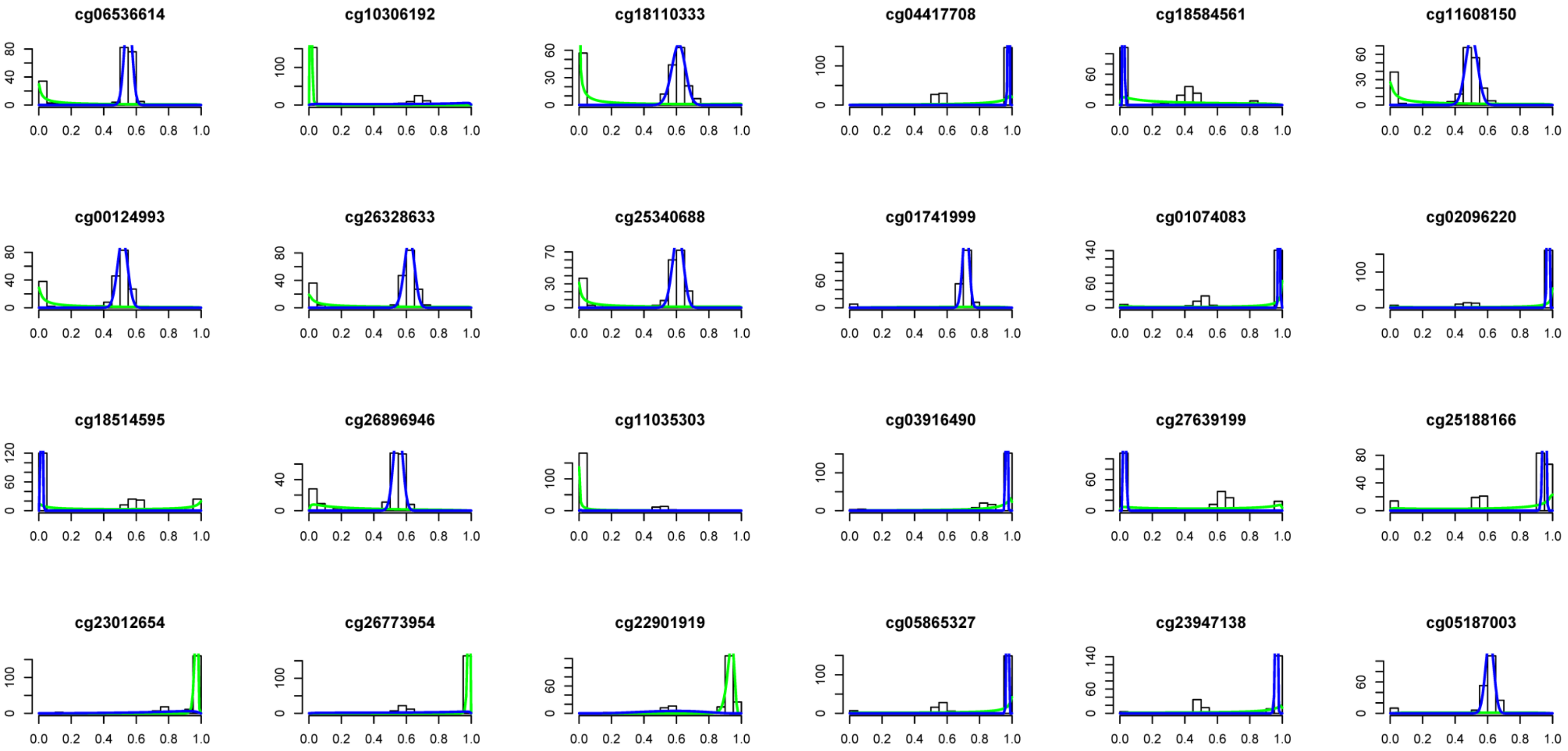

Supplementary Figure3

Histogram of the logistic-transformed M-values (which are approximately equal to % methylation values) for the 24 probes listed in Table 1, overlaid with the Mendelian model's fitted distribution for carriers (blue lines) and non-carrier (green lines).

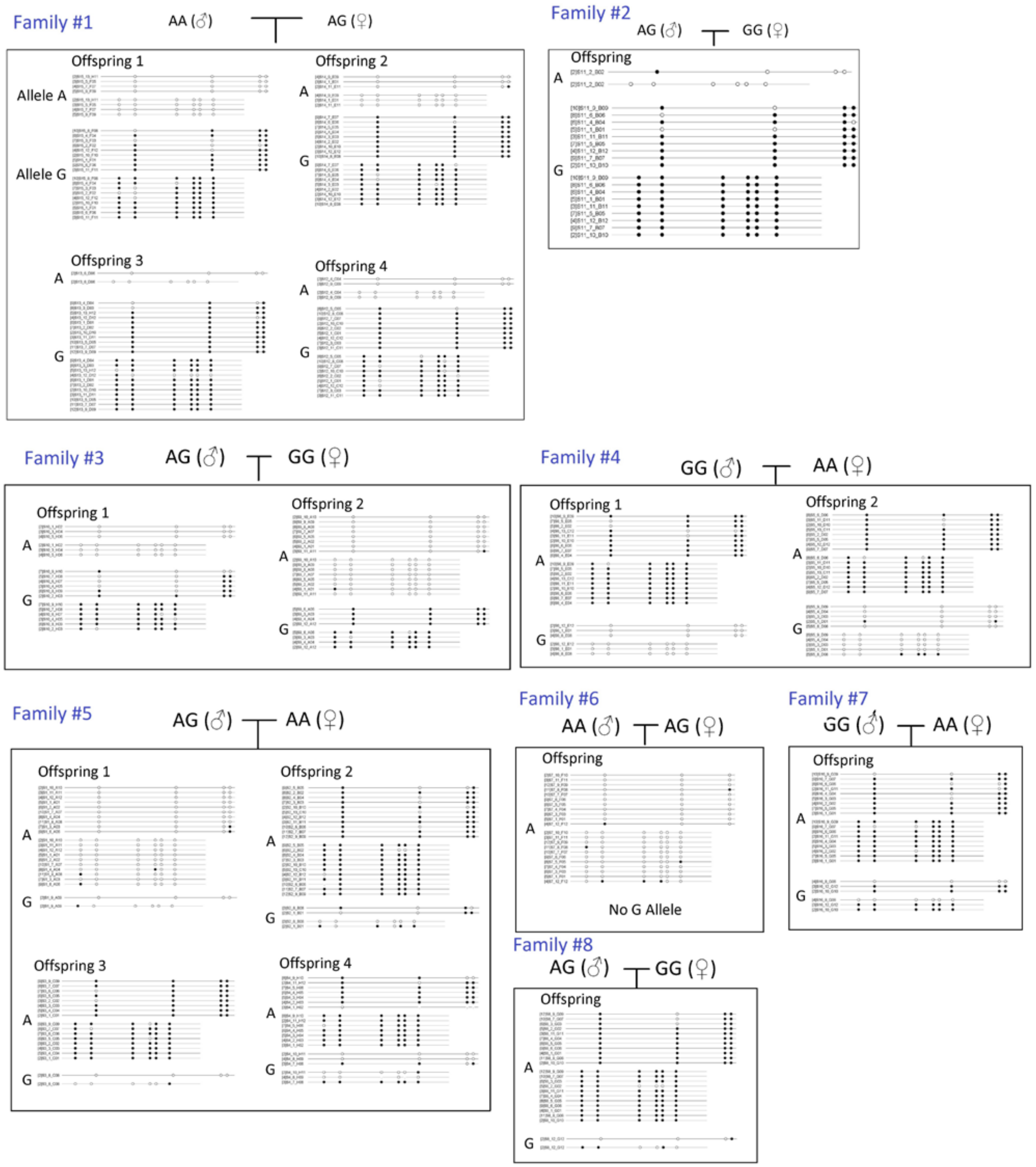

Supplementary Figure 4

Epigrams showing clonal bisulfite sequencing results at the *VTRNA2-1* locus (chr5:135415564-135415817) of blood DNA samples from 8 immediate families. Multiple offspring were assessed in 4 families. In total, methylation at 10 CpG sites from 16 offspring individuals was assessed. Filled circle indicates methylation at given CpG site. All individuals were genotyped at rs2346019 and this was used to distinguish the parent alleles in each offspring. The genotypes for each parent are shown on the top. In offspring, the bisulfite sequencing results from individual clones were separated by genotypes at this SNP. Strong allele-specific methylation (ASM) is observed in all individuals and hypermethylation of the maternally inherited allele was apparent in most individuals. One individual had obvious loss of methylation in both paternal and maternal alleles (Family #5 Offspring 1).

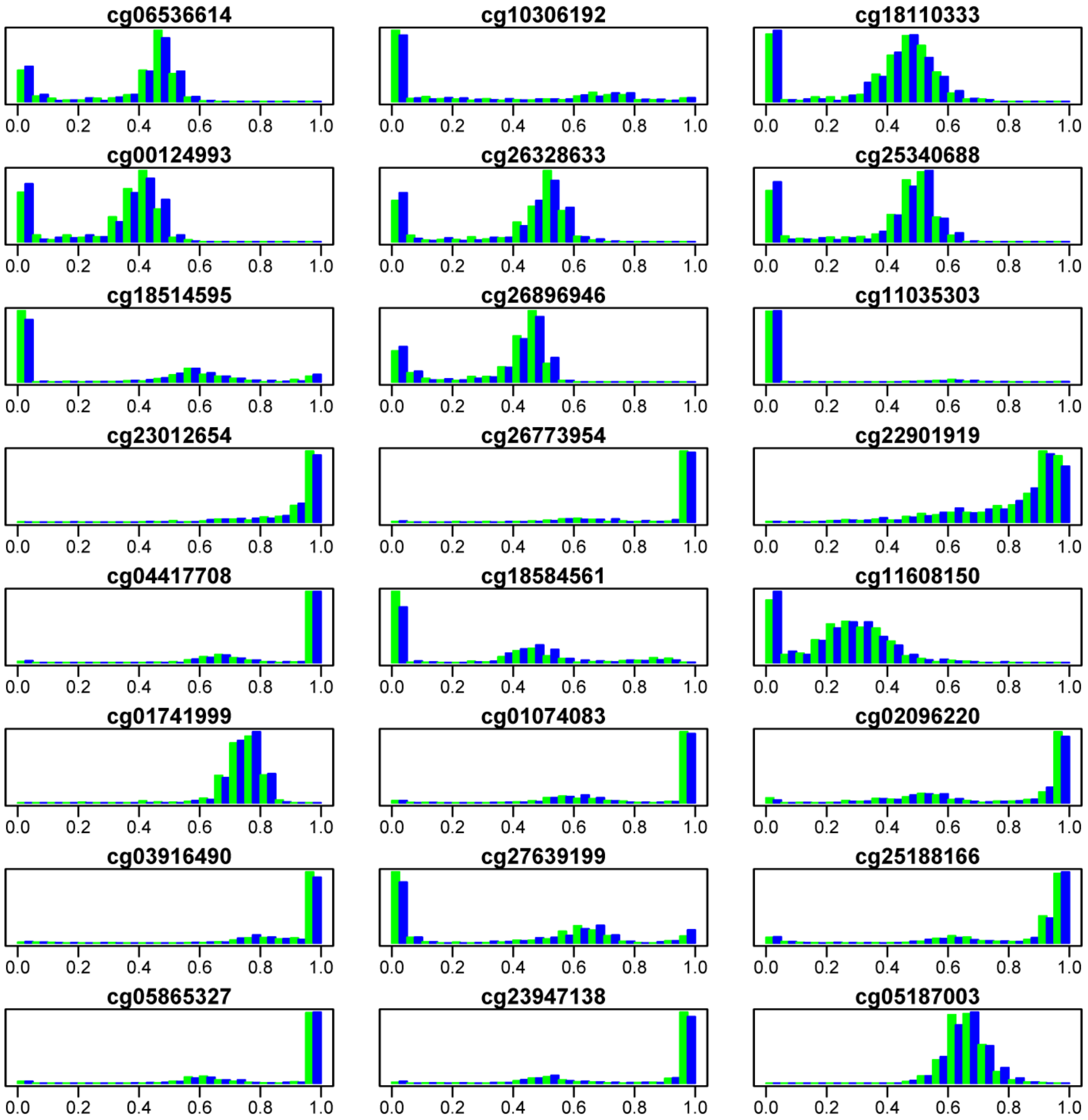

Supplementary Figure 5

Histograms for the Melbourne Collaborative Cohort Study cases (blue bars) and controls (green bars), showing the distributions of logistic-transformed M-values of the 24 heritable methylation sites that were associated with breast cancer risk in the family-based analyses.

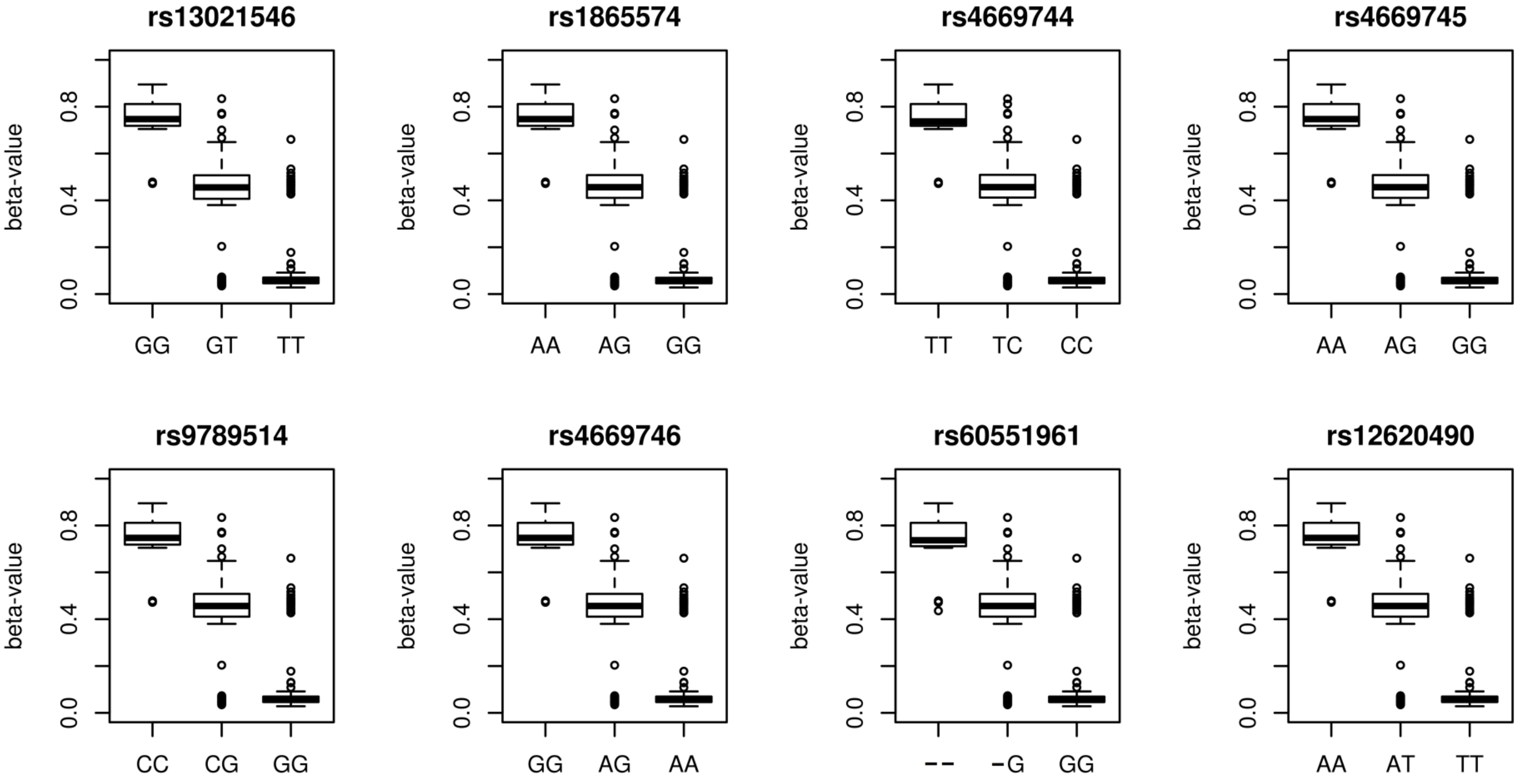

Suppementary Figure 6

Boxplots showing the association between DNA methylation and genetic variants within 1kb of cg18584561 (*GREB1)*. Imputed genotypes at 8 proximal SNPs from iCOGS (Breast Cancer Association Consortium [37] are shown on X-axis and Beta (Methylation) values are shown on Y-axis.

Supplementary Tables

Supplementary Table 1

The number of breast cancer cases and controls who are hypo-, hemi- or hypermethylated (defined by beta in 0.0-0.2, 0.2-0.8 or 0.8-1.0, respectively) for the 24 heritable probes whose carrier probabilities were found in other analyses to be associated with breast cancer, with a p-value for association between this categorised methylation variable and affected status.

Probe	Gene	Hypomethylated		Hemimethylated		Hypermethylated		p-value
		Cases	Controls	Cases	Controls	Cases	Controls	
cg06536614	MIR886 (VTRNA2-1)	15	19	68	87	0	0	1
cg10306192	MMP27	61	76	21	28	1	2	0.9
cg18110333	DUSP22	16	32	67	74	0	0	0.09
cg00124993	MIR886 (VTRNA2-1)	17	20	66	86	0	0	0.9
cg26328633	MIR886 (VTRNA2-1)	17	19	66	87	0	0	0.7
cg25340688	MIR886 (VTRNA2-1)	17	19	66	87	0	0	0.7
cg18514595	-	47	63	26	32	10	11	0.9
cg26896946	MIR886 (VTRNA2-1)	14	19	69	87	0	0	1
cg11035303	ANO10	73	93	10	13	0	0	1
cg23012654	-	2	0	19	16	62	90	0.08
cg26773954	-	2	0	20	20	61	86	0.2
cg22901919	CLGN	0	0	27	22	56	84	0.09
cg04417708	ZZEF1	0	0	21	31	62	75	0.6
cg18584561	GREB1	45	59	36	46	2	1	0.8
cg11608150	-	17	20	66	86	0	0	0.9
cg01741999	PNKD	9	2	74	104	0	0	0.01
cg01074083	XYLT1	5	3	23	27	55	76	0.5
cg02096220	-	3	4	19	19	61	83	0.7
cg03916490	C7orf50	3	5	18	22	62	79	1
cg27639199	TMC3	40	62	32	39	11	5	0.08
cg25188166	-	5	8	18	23	60	75	1
cg05865327	PPP2R5C	4	1	28	22	51	83	0.02
cg23947138	RASA3	1	1	19	27	63	78	0.9
cg05187003	IL10RB	9	1	74	105	0	0	0.005

Supplementary Table 2

The mean carrier probabilities for cases and controls, and p-value for a difference between them, for the 24 heritable probes whose carrier probabilities were found in other analyses to be associated with breast cancer.

Probe	Gene	Mean carrier probabilities (95% confidence interval) for		p-value
		Cases	Controls	
cg06536614	MIR886 (VTRNA2-1)	0.461 (0.415-0.507)	0.222 (0.202-0.243)	1.77E-11
cg10306192	MMP27	0.294 (0.253-0.334)	0.139 (0.121-0.156)	1.53E-06
cg18110333	DUSP22	0.446 (0.402-0.49)	0.204 (0.184-0.223)	1.93E-12
cg00124993	MIR886 (VTRNA2-1)	0.433 (0.389-0.477)	0.212 (0.192-0.231)	7.30E-11
cg26328633	MIR886 (VTRNA2-1)	0.432 (0.388-0.475)	0.211 (0.191-0.23)	4.78E-11
cg25340688	MIR886 (VTRNA2-1)	0.43 (0.387-0.474)	0.211 (0.192-0.231)	8.05E-11
cg18514595	-	0.404 (0.361-0.448)	0.193 (0.174-0.212)	8.32E-10
cg26896946	MIR886 (VTRNA2-1)	0.442 (0.399-0.485)	0.212 (0.193-0.231)	6.53E-12
cg11035303	ANO10	0.463 (0.422-0.505)	0.218 (0.2-0.236)	3.18E-14
cg23012654	-	0.197 (0.162-0.231)	0.083 (0.067-0.098)	8.23E-05
cg26773954	-	0.259 (0.222-0.297)	0.101 (0.085-0.118)	5.67E-07
cg22901919	CLGN	0.218 (0.182-0.253)	0.078 (0.063-0.094)	6.23E-06
cg04417708	ZZEF1	0.402 (0.361-0.442)	0.182 (0.164-0.2)	1.14E-11
cg18584561	GREB1	0.315 (0.274-0.355)	0.149 (0.132-0.167)	2.01E-07
cg11608150	-	0.395 (0.354-0.436)	0.199 (0.181-0.217)	5.62E-10
cg01741999	PNKD	0.422 (0.382-0.463)	0.214 (0.196-0.232)	3.46E-11
cg01074083	XYLT1	0.339 (0.3-0.379)	0.175 (0.158-0.193)	8.15E-08
cg02096220	-	0.37 (0.331-0.41)	0.186 (0.168-0.203)	2.44E-09
cg03916490	C7orf50	0.372 (0.331-0.413)	0.176 (0.158-0.194)	1.16E-09
cg27639199	TMC3	0.358 (0.315-0.4)	0.174 (0.156-0.193)	1.19E-07
cg25188166	-	0.344 (0.304-0.384)	0.156 (0.138-0.174)	4.83E-09
cg05865327	PPP2R5C	0.341 (0.301-0.381)	0.17 (0.152-0.187)	2.35E-08
cg23947138	RASA3	0.418 (0.376-0.459)	0.197 (0.178-0.215)	7.89E-12
cg05187003	IL10RB	0.406 (0.367-0.445)	0.204 (0.187-0.221)	2.84E-11

Supplementary Table 3

Sensitivity analyses for the associations with risk of breast cancer in the MCCS, at the 24 methylation sites showing heritable epimutations and associated with breast cancer in high-risk families.

Model 1: Adjustment for BMI (continuous), cigarette smoking (Never; Former, Current), alcohol drinking (4 categories), time between blood collection and diagnosis, sample type (DBS, PBMC, BC), and matching variables (year of birth, year of blood draw, country of birth, and sample type for the vast majority of case-control pairs)

Site	Chr.	Position	UCSC ref	OR[a]	95% CI	p
cg06536614	5	135416381	*MIR886 (VTRNA2-1)*	0.98	0.90-1.05	0.497
cg10306192	11	102576374	*MMP27*	1.03	0.98-1.08	0.235
cg18110333	6	292329	*DUSP22*	0.98	0.93-1.04	0.588
cg00124993	5	135416412	*MIR886 (VTRNA2-1)*	0.98	0.91-1.06	0.667
cg26328633	5	135416394	*MIR886 (VTRNA2-1)*	0.99	0.91-1.07	0.761
cg25340688	5	135416398	*MIR886 (VTRNA2-1)*	0.97	0.90-1.05	0.441
cg18514595	22	49579968	*unannotated*	1.05	0.99-1.11	0.077
cg26896946	5	135416405	*MIR886 (VTRNA2-1)*	0.95	0.85-1.07	0.426
cg11035303	3	43465503	*ANO10*	1.00	0.93-1.08	0.894
cg23012654	14	97493395	*unannotated*	0.97	0.88-1.06	0.503
cg26773954	13	111969980	*unannotated*	1.01	0.94-1.08	0.813
cg22901919	4	141317067	*CLGN*	0.93	0.83-1.04	0.224
cg04417708	17	4043867	*ZZEF1*	1.00	0.93-1.08	0.989
**cg18584561**	**2**	**11682017**	***GREB1***	**1.08**	**1.01-1.14**	**0.015**
cg11608150	5	135415948	*unannotated*	0.96	0.90-1.04	0.311
**cg01741999**	**2**	**219137824**	***PNKD***	**2.16**	**1.11-4.21**	**0.027**
cg01074083	16	17516862	*XYLT1*	0.99	0.93-1.06	0.749
cg02096220	4	129212177	*unannotated*	1.01	0.95-1.07	0.743
**cg03916490**	**7**	**1080558**	***C7orf50***	**0.87**	**0.78-0.97**	**0.012**
**cg27639199**	**15**	**81666528**	***TMC3***	**1.07**	**1.01-1.13**	**0.018**
cg25188166	3	119420208	*unannotated*	0.98	0.91-1.05	0.551
cg05865327	14	102274741	*PPP2R5C*	1.02	0.94-1.11	0.589
cg23947138	13	114782778	*RASA3*	0.94	0.87-1.01	0.091
cg05187003	21	34641507	*IL10RB*	0.99	0.62-1.57	0.950

Model 1 (SD). Same as Model 1 with results given per 1 Standard deviation of M-values (Table 1) + associations by breast cancer subtype

Site	Chr.	Position	UCSC ref	OR[a]	95% CI	p	ER- (N=317)		ER+ (N=106)		Heterogeneity
							OR	95% CI	OR	95% CI	p
**cg06536614**	**5**	**135416381**	***MIR886 (VTRNA2-1)***	**0.95**	**0.83-1.10**	**0.497**	**1.02**	**0.86-1.21**	**0.71**	**0.53-0.96**	**0.034**
cg10306192	11	102576374	*MMP27*	1.09	0.94-1.27	0.235	1.05	0.88-1.24	1.18	0.88-1.58	0.500
cg18110333	6	292329	*DUSP22*	0.96	0.83-1.11	0.588	1.03	0.87-1.21	0.78	0.57-1.07	0.128
cg00124993	5	135416412	*MIR886 (VTRNA2-1)*	0.97	0.84-1.12	0.667	1.01	0.85-1.20	0.77	0.57-1.04	0.110
cg26328633	5	135416394	*MIR886 (VTRNA2-1)*	0.98	0.85-1.13	0.761	1.03	0.87-1.22	0.78	0.59-1.04	0.101
cg25340688	5	135416398	*MIR886 (VTRNA2-1)*	0.95	0.82-1.09	0.441	0.99	0.84-1.17	0.76	0.57-1.03	0.132
cg18514595	22	49579968	*unannotated*	1.14	0.99-1.31	0.077	1.08	0.92-1.28	1.24	0.93-1.65	0.436
cg26896946	5	135416405	*MIR886 (VTRNA2-1)*	0.94	0.82-1.09	0.426	0.99	0.84-1.18	0.74	0.55-1.00	0.090
cg11035303	3	43465503	*ANO10*	1.01	0.88-1.16	0.894	1.07	0.91-1.25	0.90	0.65-1.23	0.336
cg23012654	14	97493395	*unannotated*	0.95	0.83-1.10	0.503	0.98	0.83-1.16	0.90	0.68-1.19	0.594
cg26773954	13	111969980	*unannotated*	1.02	0.88-1.17	0.813	0.99	0.84-1.18	1.08	0.82-1.42	0.602
cg22901919	4	141317067	*CLGN*	0.91	0.78-1.06	0.224	0.91	0.77-1.09	0.92	0.67-1.25	0.989
cg04417708	17	4043867	*ZZEF1*	1.00	0.87-1.15	0.989	1.00	0.86-1.17	1.01	0.75-1.36	0.973
**cg18584561**	**2**	**11682017**	***GREB1***	**1.18**	**1.03-1.36**	**0.015**	**1.18**	**1.01-1.38**	**1.14**	**0.86-1.51**	**0.809**
cg11608150	5	135415948	*unannotated*	0.93	0.80-1.07	0.311	0.94	0.80-1.12	0.79	0.58-1.07	0.302
**cg01741999**	**2**	**219137824**	***PNKD***	**1.26**	**1.03-1.54**	**0.027**	**1.21**	**0.97-1.50**	**1.57**	**0.96-2.57**	**0.324**
**cg01074083**	**16**	**17516862**	***XYLT1***	**0.98**	**0.84-1.13**	**0.749**	**1.08**	**0.90-1.29**	**0.72**	**0.53-0.98**	**0.021**
**cg02096220**	**4**	**129212177**	***unannotated***	**1.02**	**0.89-1.18**	**0.743**	**0.94**	**0.80-1.11**	**1.31**	**0.97-1.78**	**0.050**
**cg03916490**	**7**	**1080558**	***C7orf50***	**0.83**	**0.72-0.96**	**0.012**	**0.81**	**0.68-0.96**	**0.92**	**0.70-1.20**	**0.458**
**cg27639199**	**15**	**81666528**	***TMC3***	**1.19**	**1.03-1.36**	**0.018**	**1.18**	**1.00-1.40**	**1.18**	**0.90-1.56**	**0.996**
cg25188166	3	119420208	*unannotated*	0.96	0.83-1.10	0.551	0.98	0.83-1.15	0.92	0.71-1.20	0.714
cg05865327	14	102274741	*PPP2R5C*	1.04	0.90-1.20	0.589	1.01	0.86-1.20	1.09	0.81-1.45	0.688
**cg23947138**	**13**	**114782778**	***RASA3***	**0.89**	**0.78-1.02**	**0.091**	**0.80**	**0.68-0.94**	**1.24**	**0.93-1.65**	**0.008**
**cg05187003**	**21**	**34641507**	**IL10RB**	**1.00**	**0.86-1.15**	**0.950**	**0.99**	**0.84-1.17**	**1.03**	**0.76-1.38**	**0.839**

Model 2. Model 1 (SD) + additional adjustement for white blood cell composition (estimated with the Houseman algorithm)

Site	Chr.	Position	UCSC ref	OR[a]	95% CI	p
cg06536614	5	135416381	*MIR886 (VTRNA2-1)*	0.97	0.84-1.12	0.676
cg10306192	11	102576374	*MMP27*	1.09	0.93-1.27	0.292
cg18110333	6	292329	*DUSP22*	0.96	0.83-1.12	0.612
cg00124993	5	135416412	*MIR886 (VTRNA2-1)*	0.99	0.85-1.15	0.902
cg26328633	5	135416394	*MIR886 (VTRNA2-1)*	0.99	0.86-1.15	0.939
cg25340688	5	135416398	*MIR886 (VTRNA2-1)*	0.96	0.83-1.12	0.620
cg18514595	22	49579968	*unannotated*	1.10	0.95-1.28	0.183
cg26896946	5	135416405	*MIR886 (VTRNA2-1)*	0.96	0.83-1.11	0.608
cg11035303	3	43465503	*ANO10*	1.02	0.88-1.17	0.801
cg23012654	14	97493395	*unannotated*	0.98	0.85-1.13	0.781
cg26773954	13	111969980	*unannotated*	1.01	0.88-1.17	0.875
cg22901919	4	141317067	*CLGN*	0.95	0.81-1.12	0.576
cg04417708	17	4043867	*ZZEF1*	0.99	0.86-1.15	0.929
**cg18584561**	**2**	**11682017**	***GREB1***	**1.18**	**1.02-1.35**	**0.022**
cg11608150	5	135415948	*unannotated*	0.94	0.81-1.09	0.449
**cg01741999**	**2**	**219137824**	***PNKD***	**1.20**	**0.98-1.48**	**0.085**
cg01074083	16	17516862	*XYLT1*	0.99	0.85-1.15	0.909
cg02096220	4	129212177	*unannotated*	1.02	0.89-1.18	0.759
**cg03916490**	**7**	**1080558**	***C7orf50***	**0.81**	**0.70-0.94**	**0.006**
**cg27639199**	**15**	**81666528**	***TMC3***	**1.17**	**1.01-1.35**	**0.033**
cg25188166	3	119420208	*unannotated*	1.01	0.87-1.17	0.918
cg05865327	14	102274741	*PPP2R5C*	0.99	0.85-1.15	0.914
cg23947138	13	114782778	*RASA3*	0.89	0.77-1.02	0.085
cg05187003	21	34641507	*IL10RB*	0.95	0.82-1.10	0.504

Model 3. Model 1 + additional adjustement for age at menarche, menopausal status, number of live births, and use of hormonal replacement therapy

Site	Chr.	Position	UCSC ref	OR[a]	95% CI	p
cg06536614	5	135416381	*MIR886 (VTRNA2-1)*	0.96	0.83-1.11	0.554
cg10306192	11	102576374	*MMP27*	1.10	0.95-1.28	0.219
cg18110333	6	292329	*DUSP22*	0.98	0.85-1.14	0.831
cg00124993	5	135416412	*MIR886 (VTRNA2-1)*	0.98	0.84-1.13	0.773
cg26328633	5	135416394	*MIR886 (VTRNA2-1)*	0.99	0.85-1.14	0.849
cg25340688	5	135416398	*MIR886 (VTRNA2-1)*	0.95	0.82-1.1	0.523
cg18514595	22	49579968	*unannotated*	1.13	0.98-1.31	0.104
cg26896946	5	135416405	*MIR886 (VTRNA2-1)*	0.95	0.82-1.1	0.500
cg11035303	3	43465503	*ANO10*	1.02	0.88-1.17	0.825
cg23012654	14	97493395	*unannotated*	0.94	0.82-1.09	0.419
cg26773954	13	111969980	*unannotated*	1.03	0.89-1.19	0.728
cg22901919	4	141317067	*CLGN*	0.91	0.78-1.07	0.262
cg04417708	17	4043867	*ZZEF1*	1.01	0.88-1.17	0.838
**cg18584561**	**2**	**11682017**	***GREB1***	**1.20**	**1.04-1.38**	**0.012**
cg11608150	5	135415948	*unannotated*	0.93	0.81-1.08	0.368
**cg01741999**	**2**	**219137824**	***PNKD***	**1.23**	**1-1.51**	**0.045**
cg01074083	16	17516862	*XYLT1*	0.97	0.84-1.13	0.728
cg02096220	4	129212177	*unannotated*	1.03	0.9-1.19	0.657
**cg03916490**	**7**	**1080558**	***C7orf50***	**0.83**	**0.71-0.96**	**0.010**
**cg27639199**	**15**	**81666528**	***TMC3***	**1.18**	**1.02-1.36**	**0.024**
cg25188166	3	119420208	*unannotated*	0.96	0.83-1.11	0.562
cg05865327	14	102274741	*PPP2R5C*	1.05	0.9-1.21	0.541
cg23947138	13	114782778	*RASA3*	0.89	0.77-1.02	0.092
cg05187003	21	34641507	*IL10RB*	1.00	0.86-1.16	0.976

Supplementary Table 4
The risk of breast cancer in the general population (Melbourne Collaborative Cohort Study) calculated by carrier probabilities.

Site	Chr.	Position	UCSC ref	OR	95% CI	p
cg06536614	5	135416381	*MIR886 (VTRNA2-1)*	1.05	0.69-1.61	0.825
cg10306192	11	102576374	*MMP27*	1.09	0.79-1.49	0.604
cg18110333	6	292329	*DUSP22*	0.75	0.49-1.15	0.187
cg00124993	5	135416412	*MIR886 (VTRNA2-1)*	1.52	0.96-2.41	0.076
cg26328633	5	135416394	*MIR886 (VTRNA2-1)*	1.24	0.80-1.94	0.339
cg25340688	5	135416398	*MIR886 (VTRNA2-1)*	1.07	0.69-1.68	0.753
cg18514595	22	49579968	*unannotated*	0.68	0.49-0.94	0.018
cg26896946	5	135416405	*MIR886 (VTRNA2-1)*	1.25	0.78-2.01	0.350
cg11035303	3	43465503	*ANO10*	1.42	0.95-2.12	0.084
cg23012654	14	97493395	*unannotated*	1.14	0.80-1.64	0.472
cg26773954	13	111969980	*unannotated*	0.89	0.63-1.25	0.496
cg22901919	4	141317067	*CLGN*	1.17	0.84-1.62	0.360
cg04417708	17	4043867	*ZZEF1*	1.02	0.72-1.44	0.928
**cg18584561**	**2**	**11682017**	***GREB1***	**0.63**	**0.42-0.94**	**0.023**
cg11608150	5	135415948	*unannotated*	0.98	0.53-1.80	0.946
**cg01741999**	**2**	**219137824**	***PNKD***	**1.34**	**0.87-2.05**	**0.182**
cg01074083	16	17516862	*XYLT1*	0.92	0.65-1.31	0.639
cg02096220	4	129212177	*unannotated*	0.78	0.53-1.14	0.202
**cg03916490**	**7**	**1080558**	***C7orf50***	**0.85**	**0.59-1.23**	**0.391**
**cg27639199**	**15**	**81666528**	***TMC3***	**0.70**	**0.51-0.96**	**0.029**
cg25188166	3	119420208	*unannotated*	1.18	0.84-1.66	0.330
cg05865327	14	102274741	*PPP2R5C*	0.81	0.56-1.17	0.263
cg23947138	13	114782778	*RASA3*	0.80	0.56-1.15	0.232
cg05187003	21	34641507	*IL10RB*	0.92	0.63-1.34	0.656
