# Supplementary material for: Heritable DNA methylation marks associated with susceptibility to breast cancer
Source: Nat Commun. 2018 Feb 28;9:867. doi: 10.1038/s41467-018-03058-6 (PMC5830448; doi:10.1038/s41467-018-03058-6)
Supplement: Supplementary file 3 — Description of Additional Supplementary Files [file 41467_2018_3058_MOESM3_ESM.pdf]

Description of Additional Supplementary Files

File Name: Supplementary Data 1
Description: The 1,000 most Mendelian probes, showing the probe name, the statistic Δl, the maximized log-likelihoods l_mix and l_Mendel and the Mendelian model's maximum likelihood estimates of the M-value mean and standard deviation for non-carriers (mu0 and sd0, respectively) and carriers (mu1 and sd1, respectively).

File Name: Supplementary Data 2
Description: Families and participants included in this study. Families and individuals were deidentified.
